# PGM5: a novel diagnostic and prognostic biomarker for liver cancer

**DOI:** 10.7717/peerj.7070

**Published:** 2019-06-11

**Authors:** Yan Jiao, Yanqing Li, Peiqiang Jiang, Wei Han, Yahui Liu

**Affiliations:** 1 Department of Hepatobiliary and Pancreatic Surgery, The First Hospital of Jilin University, Changchun, China; 2 Department of Pathophysiology, College of Basic Medical Sciences, Jilin University, Changchun, China

**Keywords:** Liver cancer, Diagnosis, Prognosis, Data mining, PGM5

## Abstract

**Background:**

Liver cancer is a common malignancy and a significant public health problem worldwide, but diagnosis and prognostic evaluation remain challenging for clinicians. Metabolic reprogramming is a hallmark of cancer, and we therefore examined the diagnostic and prognostic value of a metabolic enzyme, phosphoglucomutase-like protein 5 (PGM5), in liver cancer.

**Methods:**

All data were from The Cancer Genome Atlas database. R and related statistical packages were used for data analysis. Hepatic *PGM5* expression was determined in different groups, and the chi-squared test and Fisher’s exact test were used to determine the significance of differences. The pROC package was used to determine receiver operating characteristic (ROC) curves, the survival package was used to for survival analysis and development of a Cox multivariable model, and the ggplot2 package was used for data visualization.

**Results:**

*PGM5* expression was significantly lower in cancerous than adjacent normal liver tissues, and had modest diagnostic value based on ROC analysis and calculations of area under the curve (AUC). Hepatic *PGM5* expression had positive associations with male sex and survival, but negative associations with advanced histologic type, advanced histologic grade, advanced stage, and advanced T classification. Patents with low *PGM5* levels had poorer overall survival and relapse-free survival. *PGM5* was independently associated with patient prognosis.

**Conclusion:**

*PGM5* has potential use as a diagnostic and prognostic biomarker for liver cancer.

## Introduction

Liver cancer is one of the most common malignancies, and patients typically experience poor prognoses (Llovet et al., 2016). According to global cancer statistics for 2018 (Bray et al., 2018), liver cancer is the sixth most common cancer and the fourth leading cause of cancer deaths, with about 841,000 new cases and 782,000 deaths each year. Although there have been improvements in surgical resection, transplantation, radiofrequency ablation, and chemical embolization, and therapy with sorafenib (an inhibitor of multiple tyrosine kinases) is now available, patient prognosis has only modestly improved in recent years. Histological parameters, including histological subtype and grade, together with TNM classification, are mainly used for patient evaluation and prediction of prognosis. However, accurate prediction of prognosis remains challenging for clinicians. There is an urgent need for novel biomarkers to improve diagnostic accuracy and better predict prognosis.

Metabolic reprogramming is a hallmark of all cancers. Phosphoglucomutase-like protein 5 (PGM5, also called aciculin), which metabolizes glucose-1-phosphate into glucose-6-phosphate, may play an important role in liver cancer. In the past ten years, studies of PGM5 have focused on its role in muscle tissues, and reported its associations with the cytoskeletal proteins dystrophin and utrophin (Belkin & Burridge, 1994; Belkin & Burridge, 1995a; Belkin & Burridge, 1995b). Several additional studies identified its chromosome fusion site and relationship with telomeres (Edwards et al., 1995; Fan et al., 2002). Recent studies used cell transformation to investigate its expression and pathogenic role in bladder and colorectal cancers (Li et al., 2018; Uzozie et al., 2017).

However, no studies have yet reported the clinical significance, diagnostic value, and prognostic value of PGM5 in patients with liver cancer. We examined the hepatic expression of PGM5 in patients with liver cancer, determined its association with clinical parameters, calculated its diagnostic value using receiver operating characteristic (ROC) curves, and performed survival analysis and Cox modeling to evaluate its effect on prognosis.

## Materials & Methods

### Data mining of a public database

Data mining was used to obtain raw data on patients with liver hepatocellular carcinoma. In particular, RNAseq data of PGM5 and clinical data were downloaded from The Cancer Genome Atlas database using UCSC Xena (https://xenabrowser.net/datapages/?cohort=TCGA%20Liver%20Cancer%20(LIHC)&removeHub=https%3A%2F%2Fxena.treehouse.gi.ucsc.edu%3A443%22). There was no need for ethical approval because all data were publicly available.

### Statistical analysis

All statistical analyses produce were performed using R (version 3.5.2) and related packages (R Development Core Team, 2018). *PGM5* expression data are presented in boxplots. The Wilcoxon rank-sum test (also known as Mann–Whitney non-parametric test) was used to compare two groups, and the Kruskal–Wallis test was used to compare three or more groups. ROC was drawn using the pROC package to evaluate the diagnostic value of PGM5 by calculation of the AUC (Robin et al., 2011). Patients were divided into a high expression group and a low expression group using the threshold PGM5 level identified from the ROC curve. The chi-squared test and Fisher’s exact test were used to assess the significance of associations between PGM5 level and clinical parameters. Survival analysis and the Cox model were implemented using the survival package in R to determine the prognostic value of PGM5 overall, and in different subgroups (Therneau & Grambsch, 2000), with calculations of hazard ratios (HRs) and 95% confidence intervals (95% CIs). Data were plotted using the ggplot2 package in R (Wickham, 2011).

## Results

### Characteristics of patients with hepatocellular carcinoma

The Cancer Genome Atlas database provided the characteristics of 373 patients with hepatocellular carcinoma, including age, sex, cancer stage, histologic grade, histological type, TNM classification, presence of residual tumor, and vital status (Table 1).

### Lower hepatic PGM5 expression in cancerous than normal tissues

**Figure 1:**
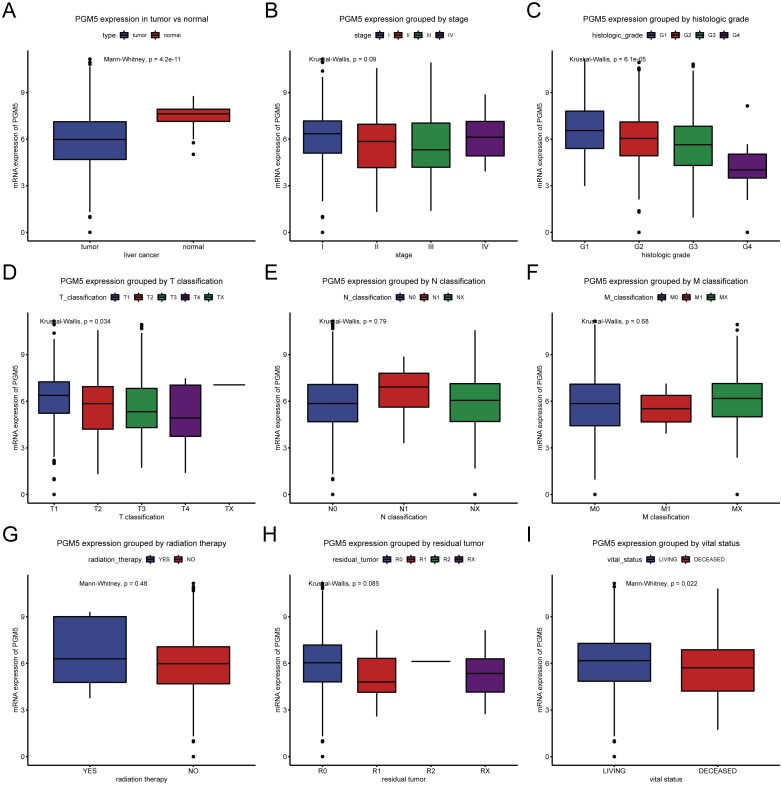
Expression of *PGM5* in liver cancer. Expression of *PGM5* in cancerous vs. adjacent normal liver tissues (A), and according to clinical stage (B), histologic grade (C), TNM classification (D–F), receipt of radiation therapy (G), presence of residual tumor (H), and survival (I). Each box plot shows the median (center line), upper and lower quartiles (box), 95% confidence intervals (vertical lines), and outliers (points).

We determined the association of *PGM5* expression with different tissue characteristics (Fig. 1). The results show that *PGM5* expression was lower in tissues with liver cancer (*n* = 373) than in adjacent normal liver tissues (*n* = 50; *P* = 4.2 ×10^−11^). In addition, *PGM5* expression had inverse correlations with advanced histologic grade (*P* = 6.1 × 10^−5^) and advanced T classification (*P* = 0.034), and a positive correlation with survival (*P* = 0.022).

### Hepatic PGM5 expression has diagnostic value in liver cancer

**Figure 2:**
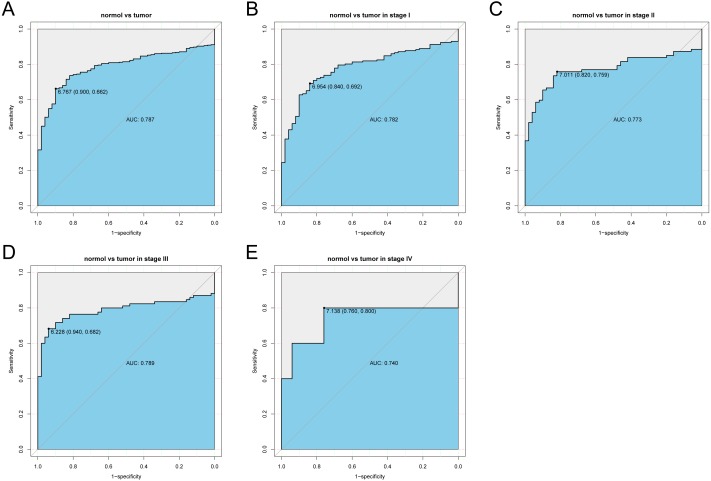
Receiver operating characteristic analysis of hepatic *PGM5* expression. Receiver operating characteristic analysis of hepatic *PGM5* expression in (A) normal vs. cancerous tissues overall (A), normal vs. stage I cancerous tissues (B), normal vs. stage II cancerous tissues (C), normal vs. stage III cancerous tissues (D), and normal vs. stage IV cancerous tissues (E).

We analyzed the *PGM5* expression data in cancerous liver tissues using ROC analysis for all patients, and for patients with different stages of cancer (Fig. 2). The results show that *PGM5* expression had a modest diagnostic value for patients overall (AUC = 0.787) and for patients with different stages of cancer (AUC_Stage__I_ = 0.782; AUC_Stage__II_ = 0.773, AUC_Stage__III_ = 0.789; AUC_Stage__IV_ = 0.740).

### Hepatic PGM5 expression correlates with several clinical parameters

We evaluated the association of *PGM5* expression with clinical parameters by dividing patients into a high expression group and a low expression group according to the threshold value identified from the ROC curve. Analysis of these data using a chi-squared test and Fisher’s exact test (Table 2) indicated that *PGM5* expression was positively associated with male sex (*P* = 0.044) and survival (*P* = 0.009), but inversely associated with advanced histologic type (*P* = 0.045), advanced histologic grade (*P* = 0.044), advanced stage (*P* = 0.008), and advanced T classification (*P* = 0.001).

### Hepatic PGM5 expression is an independent prognostic factor

**Figure 3:**
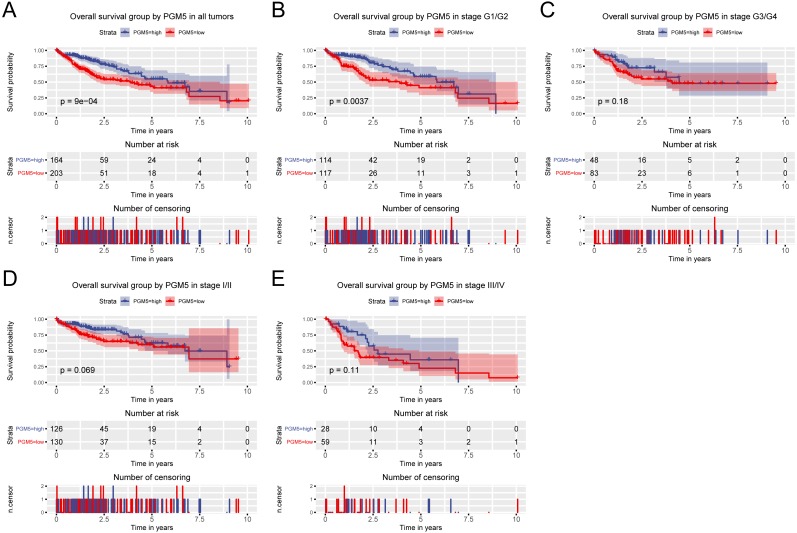
Relationship of hepatic *PGM5* expression with overall survival. Relationship of hepatic *PGM5* expression with overall survival in all patients (A), patients with histological grade G1/G2 (B), patients with histological grade G3/G4 (C), patients with clinical stage I/II (D), and patients with clinical stage III/IV (E).

**Figure 4:**
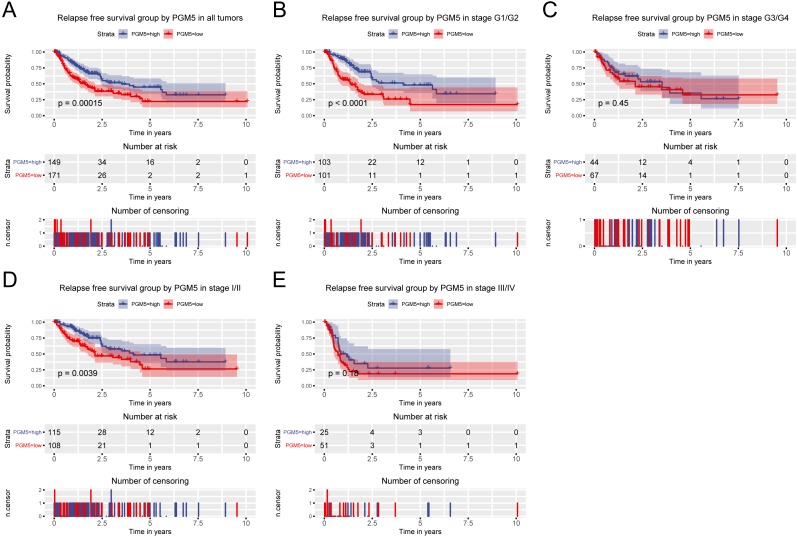
Relationship of hepatic *PGM5* expression with relapse-free survival. Relationship of hepatic *PGM5* expression with relapse-free survival in all patients (A), patients with histological grade G1/G2 (B), patients with histological grade G3/G4 (C), patients with clinical stage I/II (D), and patients with clinical stage III/IV (E).

Because high *PGM5* expression correlated with improved survival, we also examined the role of *PGM5* expression in prediction of patient prognosis. The results show that patients with lower *PGM5* expression had a shorter overall survival (OS; Fig. 3, *P* = 9 × 10^−4^) and relapse-free survival (RFS; Fig. 4, *P* = 0.00015). Subgroup analysis indicated that *PGM5* expression had significant prognostic value for OS in patients with stage I/II cancer (*P* = 0.0037) and for RFS in patients with stage I/II cancer (*P* < 0.0001) and grade G1/G2 cancer (*P* = 0.0039).

We developed a Cox model to evaluate the effect of *PGM5* expression on OS and RFS (Tables 3 and 4). The univariate Cox model indicated the variables potentially associated with *PGM5* expression were stage, histologic grade, and T classification. The multivariate Cox model identified *PGM5* expression as an independent prognostic indicator of OS (HR = 1.51, 95% CI [1.04–2.18], *P* = 0.029) and RFS (HR = 1.67, 95% CI [1.18–2.36], *P* = 0.004).

**Table 1:** Clinical characteristics of the liver cancer patients.

Characteristics	Number of patients(%)
Age	
<55	117(31.45)
≥55	255(68.55)
Gender	
FEMALE	121(32.44)
MALE	252(67.56)
histological_type	
Fibrolamellar Carcinoma	3(0.8)
Hepatocellular Carcinoma	363(97.32)
Hepatocholangiocarcinoma (Mixed)	7(1.88)
histologic_grade	
NA	5(1.34)
G1	55(14.75)
G2	178(47.72)
G3	123(32.98)
G4	12(3.22)
Stage	
NA	24(6.43)
I	172(46.11)
II	87(23.32)
III	85(22.79)
IV	5(1.34)
T_classification	
NA	2(0.54)
T1	182(48.79)
T2	95(25.47)
T3	80(21.45)
T4	13(3.49)
TX	1(0.27)
N_classification	
NA	1(0.27)
N0	253(67.83)
N1	4(1.07)
NX	115(30.83)
M_classification	
M0	267(71.58)
M1	4(1.07)
MX	102(27.35)
radiation_therapy	
NA	25(6.7)
NO	340(91.15)
YES	8(2.14)
residual_tumor	
NA	7(1.88)
R0	326(87.4)
R1	17(4.56)
R2	1(0.27)
RX	22(5.9)
Vital_status	
DECEASED	130(34.85)
LIVING	243(65.15)
Relapse	
NO	179(55.94)
YES	141(44.06)
PGM5	
High	165(44.24)
Low	208(55.76)

**Table 2:** Relationship between the clinical features and PGM5 expression in liver cancer patients.

Clinical characteristics	Variable	No. of patients	PGM5 expression				*χ*2	*p*-value
			High	%	Low	%		
Age	<55	117	53	(32.12)	64	(30.92)	0.019	0.892
	≥55	255	112	(67.88)	143	(69.08)		
Gender	FEMALE	121	44	(26.67)	77	(37.02)	4.040	**0.044**
	MALE	252	121	(73.33)	131	(62.98)		
Histological type	Fibrolamellar Carcinoma	3	3	(1.82)	0	(0)	9.357	**0.005**
	Hepatocellular Carcinoma	363	162	(98.18)	201	(96.63)		
	Hepatocholangiocarcinoma (Mixed)	7	0	(0)	7	(3.37)		
Histologic grade	G1	55	32	(19.63)	23	(11.22)	12.826	**0.004**
	G2	178	83	(50.92)	95	(46.34)		
	G3	123	47	(28.83)	76	(37.07)		
	G4	12	1	(0.61)	11	(5.37)		
Stage	I	172	91	(58.71)	81	(41.75)	11.191	**0.008**
	II	87	35	(22.58)	52	(26.8)		
	III	85	27	(17.42)	58	(29.9)		
	IV	5	2	(1.29)	3	(1.55)		
T classification	T1	182	98	(60.12)	84	(40.38)	17.897	**0.001**
	T2	95	37	(22.7)	58	(27.88)		
	T3	80	23	(14.11)	57	(27.4)		
	T4	13	4	(2.45)	9	(4.33)		
	TX	1	1	(0.61)	0	(0)		
N classification	N0	253	110	(67.07)	143	(68.75)	1.592	0.451
	N1	4	3	(1.83)	1	(0.48)		
	NX	115	51	(31.1)	64	(30.77)		
M classification	M0	267	118	(71.52)	149	(71.63)	0.631	0.809
	M1	4	1	(0.61)	3	(1.44)		
	MX	102	46	(27.88)	56	(26.92)		
Radiation therapy	NO	340	150	(97.4)	190	(97.94)	0.000	1.000
	YES	8	4	(2.6)	4	(2.06)		
Residual tumor	R0	326	151	(93.21)	175	(85.78)	5.447	0.115
	R1	17	5	(3.09)	12	(5.88)		
	R2	1	0	(0)	1	(0.49)		
	RX	22	6	(3.7)	16	(7.84)		
Vital status	DECEASED	130	45	(27.27)	85	(40.87)	6.900	**0.009**
	LIVING	243	120	(72.73)	123	(59.13)		

**Notes.**

Bold values of *P* ≤ 0.05 indicate statistically significant.

**Table 3:** Univariate analysis and multivariate analysis of liver cancer patients’ overall survival.

Parameters	Univariate analysis			Multivariate analysis		
	Hazard Ratio	95% CI (lower∼ upper)	*P* value	Hazard Ratio	95% CI (lower-upper)	*P* value
Age	1.00	0.69–1.45	0.997			
Gender	0.80	0.56–1.14	0.220			
Histological type	0.99	0.27–3.66	0.986			
Histologic grade	1.04	0.84–1.3	0.698			
Stage	1.38	1.15–1.66	0.001	0.87	0.7–1.09	0.220
T classification	1.66	1.39–1.99	0.000	1.77	1.39–2.24	0.000
N classification	0.73	0.51–1.05	0.086			
M classification	0.72	0.49–1.04	0.077			
Radiation therapy	0.51	0.26–1.03	0.060			
Residual tumor	1.42	1.13–1.8	0.003	1.39	1.09–1.78	0.008
PGM5	1.83	1.27–2.63	0.001	1.51	1.04–2.18	0.029

**Table 4:** Univariate analysis and multivariate analysis of liver cancer patients’ relapse-free survival.

Parameters	Univariate analysis			Multivariate analysis		
	Hazard Ratio	95% CI (lower∼ upper)	*P* value	Hazard Ratio	95% CI (lower-upper)	*P* value
Age	0.90	0.63–1.28	0.550			
Gender	0.99	0.7–1.41	0.966			
Histological type	2.02	0.66–6.24	0.220			
Histologic grade	0.98	0.8–1.21	0.883			
Stage	1.66	1.38–1.99	0.000	1.14	0.88–1.48	0.326
T classification	1.78	1.49–2.12	0.000	1.57	1.19–2.05	0.001
N classification	0.97	0.67–1.4	0.874			
M classification	1.17	0.79–1.74	0.432			
Radiation therapy	0.74	0.26–2.16	0.584			
Residual tumor	1.28	1.01–1.61	0.042	1.32	1.04–1.67	0.023
PGM5	1.92	1.36–2.7	0.000	1.67	1.18–2.36	0.004

## Discussion

Our team have been engaged in exploring the novel cancer biomarks for a long time (Jiao et al., 2018; Jiao et al., 2019a; Jiao et al., 2019b). The present study indicated that *PGM5* expression was lower in cancerous than adjacent normal liver tissues. Hepatic *PGM5* expression was also positively associated with male sex and survival, and negatively associated with advanced histologic type, histologic grade, clinical stage, and T classification. We also found that hepatic *PGM5* expression had significant value as a diagnostic indicator of liver cancer and that patients with low hepatic *PGM5* expression had poorer prognosis, in terms of OS and RFS. The results of our Cox model analysis indicated low hepatic *PGM5* expression was an independent indicator of poor prognosis.

PGM5 (initially named aciculin) is a cytoskeletal protein present in smooth muscle tissues (Belkin & Burridge, 1994). Initial studies reported changes of PGM5 expression during muscledifferentiation, in that there is upregulation during muscle development, and that this protein is a useful marker of the contractile/differentiated smooth muscle phenotype (Belkin & Burridge, 1994; Moiseeva & Critchley, 1997). However, little is known about the expression of PGM5 during cancer pathogenesis. The present study indicated that *PGM5* had lower expression in cancerous liver tissues than adjacent normal tissues, similar to the results of a previous study of colorectal cancer (Uzozie et al., 2017). Furthermore, we determined that hepatic *PGM5* expression had a modest diagnostic value for liver cancer overall and for each of the four stages of liver cancer, suggesting it has potential use as a novel diagnostic biomarker.

Low hepatic *PGM5* expression is associated with poor prognosis in patients with liver cancer. Previous studies found that PGM5 functions in multiple cell–matrix adherens junctions in association with dystrophin and utrophin, and that it interacts with filamin C and Xin during myofibril assembly, remodeling, and maintenance (Belkin & Burridge, 1995a; Belkin & Burridge, 1995b; Molt et al., 2014; Wakayama et al., 2000). Its chromosome fusion site is close to the telomere, and is related to the rearrangement of subtelomeric and pericentromeric regions (Fan et al., 2002; Wong et al., 2004). These previous findings suggest this protein has a role in cell–matrix adherens junctions and the regulation of telomeres during cancer progression, although no previous study has yet directly examined the specific function of PGM5 during the pathogenesis of cancer. Our study of patients with liver cancer indicated that hepatic *PGM5* expression was positively associated with male sex and survival, and negatively associated with advanced histologic type, histologic grade, stage, and T classification. Our survival analysis indicated that low hepatic *PGM5* expression was associated with poor prognosis, and was an independent prognostic factor for poor OS and RFS. These results indicate that PGM5 has potential as a prognostic biomarker, as well as a diagnostic marker, for liver cancer.

This study and several previous studies suggest that PGM5 has a role in the pathogenesis of several cancers, and has potential value as a diagnostic and prognostic biomarker for liver cancer. However, this study is based on data mining of a single public database, so our findings require verification in different populations. Our future studies will examine the role of PGM5 in liver cancer of different populations and will also examine its molecular function using *in vivo* and *in vitro* experiments.

## Conclusions

In summary, we found that *PGM5* expression was lower in cancerous than adjacent normal liver tissues, and was positively associated with male sex and survival, and negatively associated with advanced histologic type, histologic grade, stage, and T classification. In addition, patients with low expression of hepatic *PGM5* had poorer OS and RFS. Our Cox model results indicated that hepatic *PGM5* expression was an independent prognostic factor. However, our results are based on data mining of a selected group of patients from a public database, so verification is required for additional populations.

##  Supplemental Information

10.7717/peerj.7070/supp-1 Supplemental Information 1 PGM5 expression and clinical data of liver cancer extracted from TCGA database

10.7717/peerj.7070/supp-2 Supplemental Information 2 PGM5 expression in normal tissue extracted from TCGA and GTEx database

10.7717/peerj.7070/supp-3 Supplemental Information 3 Demographics for PGM5 expression in normal tissue

## Abbreviations

95% CI: 95% confidence interval
AUC: area under curve
HR: hazard ratio
OS: overall survival
PGM5: phosphoglucomutase-like protein 5
RFS: relapse-free survival
ROC: receiver operating characteristic

## Additional Information and Declarations

### Data Availability

The following information was supplied regarding data availability: The raw data are available as Supplemental Files. The data was collected from TCGA (https://cancergenome.nih.gov/) and UCSC Xena (https://xenabrowser.net/datapages/).
